# A case of cervical esophageal duplication cyst in a newborn infant

**DOI:** 10.1186/s40792-016-0157-2

**Published:** 2016-04-01

**Authors:** Shoko Kawashima, Osamu Segawa, Shuri Kimura, Masayoshi Tsuchiya, Nobuhide Henmi, Hisaya Hasegawa, Mariko Fujibayashi, Yoshihiko Naritaka

**Affiliations:** Department of Surgery, Tokyo Women’s Medical University Medical Center East, 2-1-10 Nishi Ogu, Arakawa-ku, Tokyo, 116-8567 Japan; Department of Pediatric Surgery, Tokyo Women’s Medical University, 8-1 Kawada-cho, Shinjuku-ku, Tokyo, Japan; Division of Neonatal Intensive Care, Tokyo Women’s Medical University Medical Center East, 2-1-10 Nishi Ogu, Arakawa-ku, Tokyo, 116-8567 Japan; Department of Diagnostic Pathology, Tokyo Women’s Medical University Medical Center East, 2-1-10 Nishi Ogu, Arakawa-ku, Tokyo, 116-8567 Japan

**Keywords:** Congenital duplication cyst, Esophagus, Cervical, Newborn, Simple cyst

## Abstract

Esophageal duplication cyst is a rare congenital anomaly resulting from a foregut budding error during the fourth to sixth week of embryonic development. Cervical esophageal duplication cysts are very rare and may cause respiratory distress in infancy. A full-term newborn girl who was born by normal delivery was transferred to our hospital because of swelling of the right anterior neck since birth. Cervical ultrasonography showed a 40 × 24 × 33 mm simple cyst on the right neck. Tracheal intubation was required at 2 weeks of age because of worsening external compression of the trachea. Fine-needle aspiration cytology revealed the existence of ciliated epithelium. At 1 month of age, exploration was performed through a transverse neck incision. The cyst had a layer of muscle connected to the lateral wall of the esophagus. Histopathological diagnosis was a cervical esophageal duplication cyst. We describe the clinical features of infantile cervical esophageal duplication cysts based on our experience of this rare disease in a neonate, along with a review of 19 cases previously reported in literature.

## Background

Esophageal duplication cysts are rare congenital cystic masses resulting from an error in foregut budding in the developing embryo and with a reported incidence of 1 in 8200 autopsies [1]. Most esophageal duplication cysts are in the thorax; involvement of the cervical region is distinctly rare. We report the case of a neonate with cervical esophageal duplication cyst at birth and describe the features of infantile cervical esophageal duplication cysts along with 19 infantile case reports.

## Case presentation

**Fig. 1:**
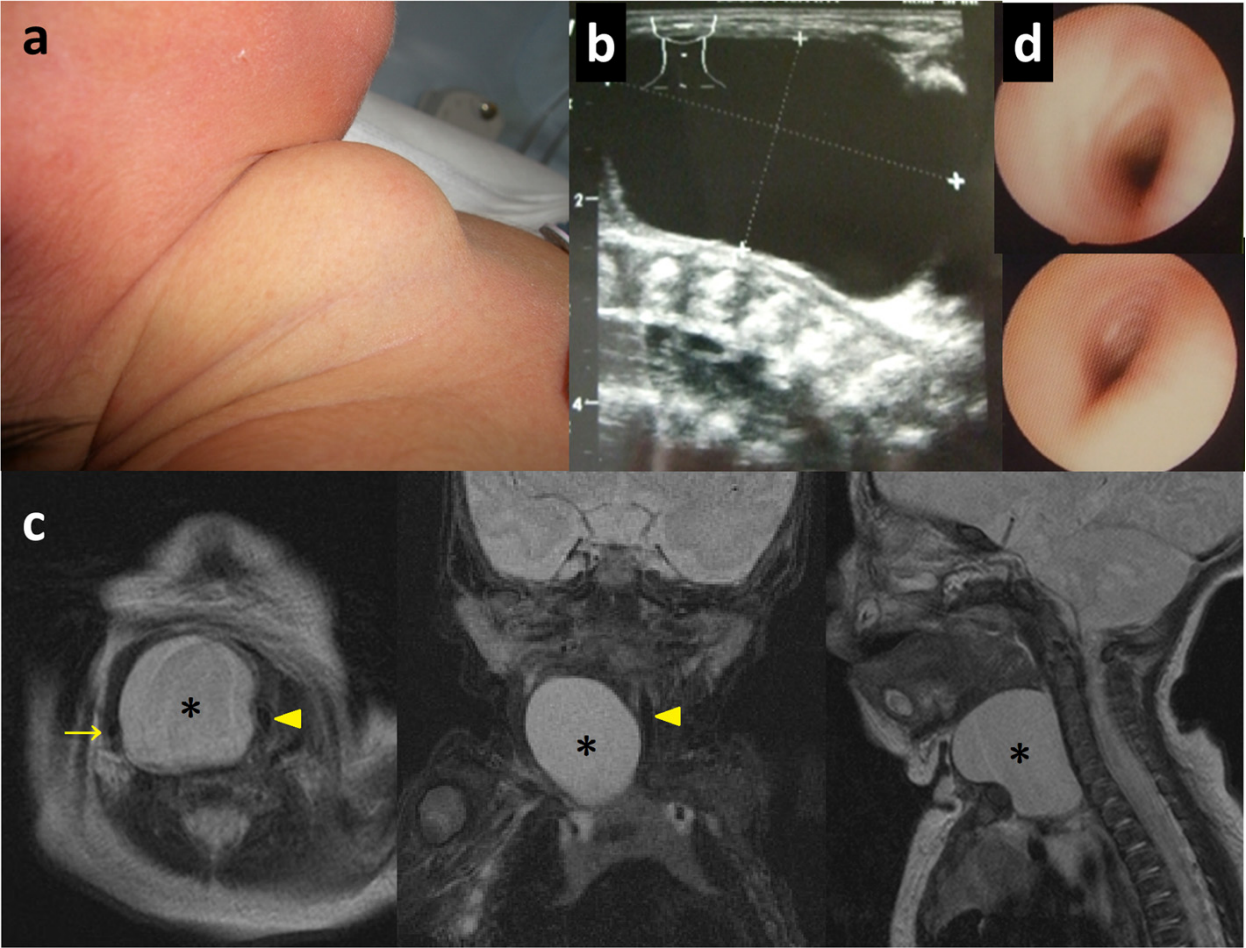
Clinical findings in a newborn infant girl with esophageal duplication cyst. **a** On physical examination, there was anterior neck swelling on the right. **b** Ultrasonography showed a simple cyst measuring 40 × 24 × 33 mm in the right neck. **c** T2-weighted enhanced magnetic resonance imaging revealed a simple cyst (*asterisk*) that displaced the sternocleidomastoid muscle anteriorly, the carotid sheath (*arrow*) to the right, and the larynx and trachea (*arrow head*) to the left. **d** Bronchoscopy showed compressive deformation of the trachea from the subglottis to the carina

A full-term newborn girl was transferred and admitted to our hospital because of swelling of the right anterior neck since birth (Fig. 1a). She did not manifest with respiratory distress and feeding difficulty on admission.

Cervical ultrasonography (Fig. 1b) showed a simple cyst measuring 40 × 24 × 33 mm on the right neck. Magnetic resonance imaging (MRI) showed a simple cyst that displaced the sternocleidomastoid muscle anteriorly, the carotid sheath to the right, and the larynx and trachea to the left (Fig. 1c). The distal edge of the cyst was attached to the thymus.

Bronchoscopy (Fig. 1d) demonstrated extrinsic compression of the trachea from the subglottis to the carina, which eventually worsened and necessitated airway control with nasotracheal intubation at 2 weeks of age. Fine-needle aspiration cytology of the cyst revealed the existence of ciliated epithelium, suggesting a bronchogenic cyst.

Barium esophagography did not show stenosis, deformity, or tracheoesophageal fistula.

**Fig. 2:**
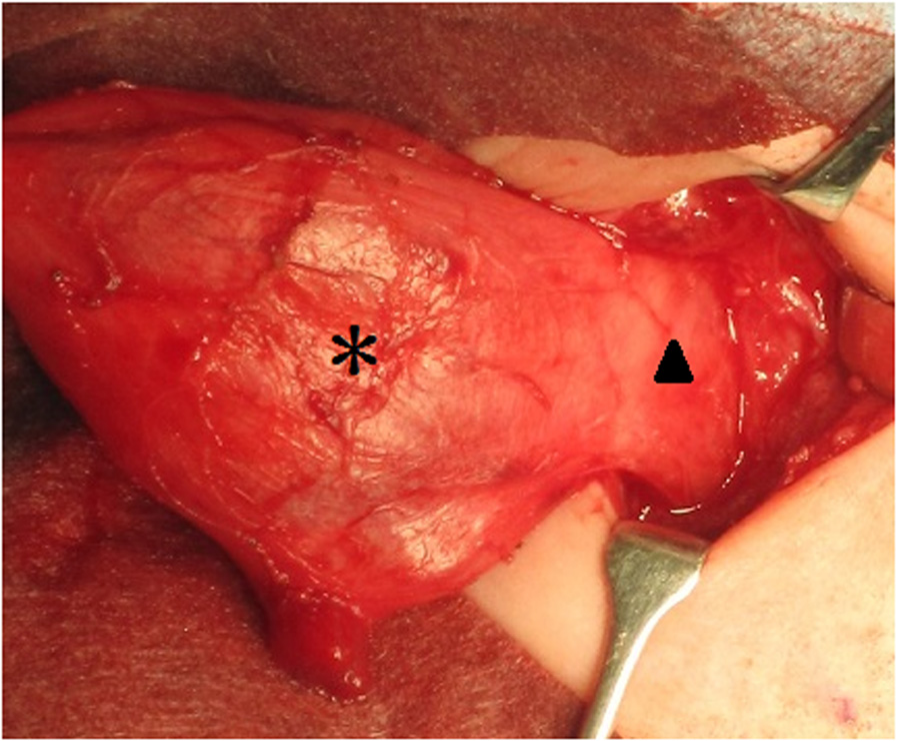
Intraoperative findings in an infant girl who underwent surgical exploration of an esophageal duplication cyst at 1 month of age. The cyst was separated from the right carotid artery and vein, thymus, vertebrae, and trachea. The cyst (*asterisk*) had a layer of muscle and was connected with the lateral wall of the esophagus (*arrow head*)

Exploration was performed through a transverse neck incision at 1 month of age (Fig. 2). Adhesiolysis of the cyst from the right carotid artery and vein, thymus, vertebrae, and trachea was performed without perforation. The cyst had a layer of muscle and was connected to the esophageal muscle layer. Therefore, we considered that it was an esophageal duplication cyst. Incision of the muscle layer was made slightly lateral to the border of the cyst and esophagus to avoid injury of the esophageal mucosa. The muscle layer of the esophagus was repaired. There was no communication between the cyst and the esophageal lumen. We had imaged the vagus nerve was around the cyst; however, the vagus nerve was not detected during operation as a result of deviating from normal position.

**Fig. 3:**
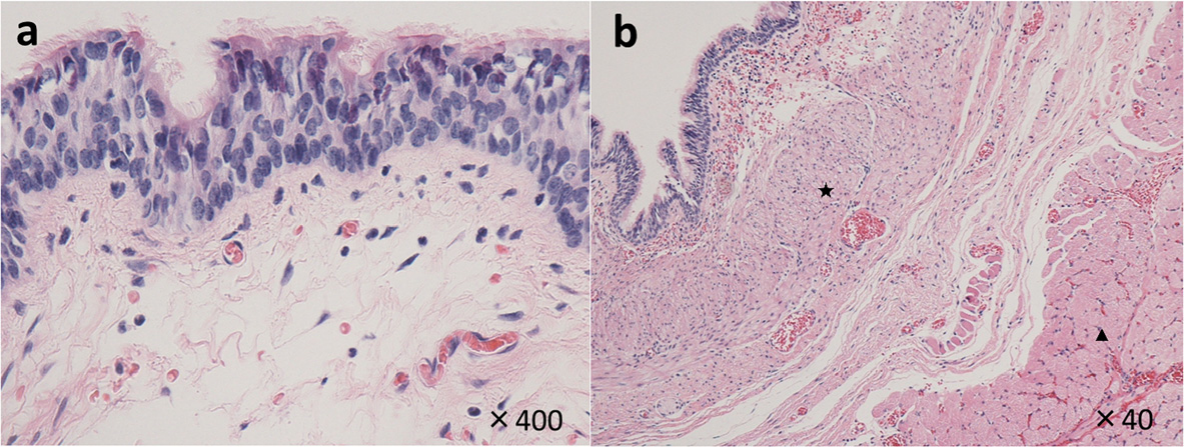
Photomicrographs of a surgically resected infantile esophageal duplication cyst. **a** Pathologic examination showed an internal layer with pseudostratified ciliated epithelium (hematoxylin and eosin stain ×400) with **b** covering layers of smooth muscle (*star*) and striated muscle (*arrow head*) (hematoxylin and eosin stain ×40)

Pathologic examination of the cyst showed an internal layer with pseudostratified ciliated epithelium (Fig. 3a) with covering layers of smooth muscle and striated muscle (Fig. 3b). The whole cyst was covered by smooth muscle layer, and about two-thirds of the cyst was covered by striated muscle layer. The luck of the striated muscle layer was the bottom of the cyst, contiguous with the thymus and innominate artery. Bronchial gland and cartilage were not present. A final diagnosis of cervical esophageal duplication cyst was made.

Extubation was carried out the day after the operation. Repeat bronchoscopy showed disappearance of the compressive deformation of the trachea. Transient right recurrent laryngeal nerve palsy was observed and controlled by nasal direct positive airway pressure for 3 weeks.

Postoperative barium esophagography showed good peristalsis without any extravasation or stricture. The patient has been doing well for 4 years now.

### Discussion

Esophageal duplication cysts are rare congenital cystic masses resulting from a foregut budding error during the fourth to sixth week of embryonic development [2]. The primitive foregut divides into dorsal and ventral segments; the dorsal segment ultimately differentiates into the esophagus, whereas the ventral segment develops into the tracheobronchial tree [3, 4].

Foregut budding error may lead to bronchogenic cyst, esophageal duplication cyst, or “bronchopulmonary foregut malformations” (BPFM), a term proposed in 1968 by Gerle et al. [5] to encompass the full spectrum of developmental aberrations of the embryonic foregut. The presence of an accessory lung bud distal to the normal lung bud has been proposed as the underlying basis of BPFM [6]. An interesting theory is that all BPFMs belong to a homogeneous group with a common embryologic pathogenesis. This group includes pulmonary sequestration, tracheoesophageal fistula, intestinal duplication cysts, neurenteric cysts, systemic arterializations of the lung, bronchogenic cysts, esophageal diverticula, and ectopic bronchial mucosal rests in the esophageal wall [7].

Esophageal duplication cyst is one of the gastrointestinal duplications that are rare congenital malformations occurring anywhere from the mouth to the anus. About 10 to 15 % of all duplication cysts in the gastrointestinal tract are esophageal [7]. In a review of 49,196 autopsies, the incidence of esophageal duplication cyst was 1:8200 [1]. The location of the cysts in the esophagus was in the lower third in 60 %, in the middle third in 17 %, and in the upper third in 23 % [1, 8].

Even among cervical cysts, esophageal duplication cysts are quite rare. As for the differential diagnosis, Hsieh et al. reported on 331 pediatric patients diagnosed as having cervical cysts [9]. From the results of histology, thyroglossal duct cysts accounted for 54.68 % of all cases, followed by cystic hygromas (25.08 %), branchial cleft cysts (16.31 %), bronchogenic cysts (0.91 %), and thymic cysts (0.30 %). Nine cases (2.72 %) remained unclassified. There was no cervical esophageal duplication cyst among the 331 infants.

In 1937, Ladd suggested the use of the term “duplications of the alimentary tract” and applied the term to congenital lesions having three characteristics: (1) the presence of a well-developed coat of smooth muscle, (2) an epithelial lining representing some type of intestinal tract mucosa, and (3) intimate anatomic association with some portion of the gastrointestinal tract [10].

The histopathological criteria for classifying a foregut duplication cyst as an esophageal cyst were developed by Arbona et al. [1] as follows:

1. The cyst is within or attached to the esophageal wall.
2. It is covered by two muscle layers.
3. The lining is squamous, columnar, cuboidal, pseudostratified, or ciliated epithelium.

In contrast to esophageal cysts, bronchogenic cysts are diagnosed based on the presence of cartilage [2, 3, 11]. Differentiating one from the other and making a definitive diagnosis before operation are difficult.

The definitive treatment for cervical esophageal duplication cysts is surgical removal, and it has been reported that there is no recurrence after exploration. Total exploration is important for suspected esophageal duplication cyst because of a possibility of coexistent squamous cell carcinoma [1, 12].

Twenty case reports of infantile cervical esophageal duplication cysts, including our own, were found upon review of English literature (Table 1) [13–29]. Our case was the sixth among newborns. Of note, 12 patients presented with signs of respiratory distress, such as stridor, in infancy; two presented with feeding difficulty; five presented with fever and/or respiratory infection; and three had asymptomatic neck swelling on admission. One case had concomitant tracheoesophageal fistula [18]. Ultrasonography, computed tomography, and MRI revealed tracheal deviation in 19 cases. Respiratory distress was caused by tracheal compressive deformation and deviation in 12 cases (60 %), three of which needed tracheal intubation. Respiratory symptoms were the most common symptoms of cervical esophageal duplication cysts in children. Consequently, these cysts should be included in the differential diagnosis of an infant with airway stenosis. Communication with the esophagus was present in four cases (20 %); this frequency was more than the previously reported 10 % [7, 30]. Antenatal ultrasonography detected the cervical esophageal duplication cysts in two cases, underscoring the value of this prenatal test.

**Table 1:** A literature review of infantile cases of cervical esophageal duplication cyst

Number	Age	Sex	Respiratory distress	Poor feeding	Fever and/or respiratory infection	Palpable mass	Tracheal deviation	Others	Antenatal US detection	Communication with esophagus	Size (cm)	Surgical intervention	Author (year)
1	25 days	M	+			+	+					Tracheostomy	Bishop and Koop (1964) [13]
												Resection at a later time	
2	6 years	M	−	−	−	+	+			−	5	A collar incision	Gans (1968) [14]
3	2 months		+	−	+		+			−	4 × 5 × 3	Transverse neck incision	Gans (1968) [14]
4	3.5 months	M	+			+	+			−		Transverse neck incision	Winslow (1984) [15]
5	9 days	M	+				+	Resuscitation, intubation		−		Transverse neck incision	Winslow (1984) [15]
6	9 months	F	−	+	+	+	+			+	3 × 4 × 6.5		Rhee (1988) [16]
7	18 months	F	−		−	+	+			−	4 × 3	Transverse neck incision	Billmire (1995) [17]
8	22 months	F	+		+	+	+			−	4		Barzilai (1995) [18]
9	0 day	M						Esophageal web, TEF		+		Neck incision	Snyder (1996) [19]
												Right thoracotomy	
10	2 years	F	+		−	− → +	+		24 weeks: cystic lesion		5 × 5	Transverse neck incision	McCullagh (2000) [20]
									32 weeks: not detected				
11	0 day	F	+			+	+	Intubation (DOL2)	−	+	6.2 × 3.4 × 0.5		Wootton-Gorges (2002) [21]
12	1 year	M			+		+	Bronchogenic cyst		−		Left thoracotomy, transverse neck incision	Yasufuku (2003) [22]
							^a^	Sequestration					
13	13 months	M	+	+			+			−	1.5 × 2.0	Neck incision	Moulton (2005) [23]
14	3 years	M	−	−	−	+	+	Drooping of the eyelid			5	Transverse neck incision	Sharma (2005) [24]
15	22 weeks G/A	M	−	−			+				1 × 0.8 × 1.5	(Resection at 6 month planned)	Sherer (2009) [25]
16	9.5 months	M	+				+				2.8 × 2.4 × 1.2	Right thoracotomy	Nayan (2010) [26]
17	6 months	M	+	−	−	−	+				2 × 2 × 2	Skin crease neck incision	Gupta (2010) [27]
18	3 months	M	+	–	+		+			+	6 × 2	Skin crease neck incision	V. Kumar (2010) [28]
19	3 months	F	–	–			+		+	–	2.5 × 2.2 × 4.5	Thoracoscopic resection	S.Y Lee (2013) [29]
20	0 day	F	− → +	− → ^b^	−	+	+	Intubation (DOL14)		−	4 × 2.4 × 3.3	Transverse neck incision	Present case

*G/A* gestational age, *F* female, *M* male, *TEF* tracheoesophageal fistula, *DOL* days of life, *US* ultrasonography

^a^By bronchogenic cyst

^b^Tube feeding

## Conclusions

This report described a very rare case of a neonate with cervical esophageal duplication cyst that was found at birth. Airway stenosis was progressive and required airway control with tracheal intubation. Exploration at 1 month of age led to the resolution of airway stenosis.

According to 19 case reports and our own, respiratory distresses caused by external compression and deviation of the trachea commonly occur in infantile cervical esophageal duplication. We assume that cervical esophageal duplication cysts should be included in the differential diagnosis of an infant with respiratory distress and airway stenosis. Definitive diagnosis is based on histopathological findings, and surgical removal of the cyst is the treatment of choice.

## Consent

Written informed consent was obtained from the patient for publication of this case report and any accompanying images. A copy of the written consent is available for review by the Editor-in-Chief of this journal.

## Abbreviations

BPFM: bronchopulmonary foregut malformations
MRI: magnetic resonance imaging
